# Dopamine-Induced Plasticity, Phospholipase D (PLD) Activity and Cocaine-Cue Behavior Depend on PLD-Linked Metabotropic Glutamate Receptors in Amygdala

**DOI:** 10.1371/journal.pone.0025639

**Published:** 2011-09-27

**Authors:** Balaji Krishnan, Kathy M. Genzer, Sebastian W. Pollandt, Jie Liu, Joel P. Gallagher, Patricia Shinnick-Gallagher

**Affiliations:** 1 Department of Pharmacology and Toxicology, University of Texas Medical Branch at Galveston, Galveston, Texas, United States of America; 2 Department of Anesthesiology, Presbyterian Hospital, Columbia University, New York, New York, United States of America; Institut National de la Santé et de la Recherche Médicale, France

## Abstract

Cocaine-cue associations induce synaptic plasticity with long lasting molecular and cellular changes in the amygdala, a site crucial for cue-associated memory mechanisms. The underlying neuroadaptations can include marked alterations in signaling via dopamine (DA) receptors (DRs) and metabotropic glutamate (Glu) receptors (mGluRs). Previously, we reported that DR antagonists blocked forms of synaptic plasticity in amygdala slices of Sprague-Dawley rats withdrawn from repeated cocaine administration. In the present study, we investigated synaptic plasticity induced by exogenous DA and its dependence on mGluR signaling and a potential role for phospholipase D (PLD) as a downstream element linked to mGluR and DR signaling. Utilizing a modified conditioned place preference (CPP) paradigm as a functional behavioral measure, we studied the neurophysiological effects after two-weeks to the last cocaine conditioning. We recorded, electrophysiologically, a DR-induced synaptic potentiation in the basolateral to lateral capsula central amygdala (BLA-lcCeA) synaptic pathway that was blocked by antagonists of group I mGluRs, particularly, the PLD-linked mGluR. In addition, we observed 2–2.5 fold increase in PLD expression and 3.7-fold increase in basal PLD enzyme activity. The enhanced PLD activity could be further stimulated (9.3 fold) by a DA D1-like (D1/5R) receptor agonist, and decreased to control levels by mGluR1 and PLD-linked mGluR antagonists. Diminished CPP was observed by infusion of a PLD-linked mGluR antagonist, PCCG-13, in the amygdala 15 minutes prior to testing, two weeks after the last cocaine injection. These results imply a functional interaction between D1/5Rs, group I mGluRs via PLD in the amygdala synaptic plasticity associated with cocaine-cues.

## Introduction

Drug addiction can be classified as a disease of learning and memory [1]. Bouts of abstinence interrupted by drug use characterize cocaine abuse [2]. Such psychostimulant abuse results from cue-associated memory mechanisms reinforced by regular drug intake [1]–[4]. Consequently, the cues associated with repeated drug exposure, and in the absence of the drug, can elicit intense craving [5]–[7] that ultimately result in relapse to drug taking. For this reason, a greater understanding of the associative learning processes that maintain the addictive state is necessary for successful treatment of cocaine addiction.

Specific amygdala subnuclei are involved with drug-cue associated memory mechanisms [6], [8]–[13]. Lesioning or inactivation of the basolateral amygdala (BLA) prevents the acquisition and expression of conditioned-cue responses associated with cocaine-seeking behavior [14]–[18] whereas inactivation of the central amygdala (CeA) alone disrupts expression but not acquisition [19]. Thus, BLA-CeA synaptic pathway is important for the expression of conditioned responses to cocaine.

Conditioned place preference (CPP) is a classical conditioning paradigm [20] wherein drug pairing to cued sensory and contextual stimuli can be quantified to study drug-cue associations [21]. CPP has also been effective in studying the contribution of specific amygdala subnuclei in acquisition and expression of conditioned responses to cocaine [22]. For example, BLA lesions prior to cocaine CPP training disrupt acquisition, while post-conditioning lesions disrupt extinction [23]. Another example illustrates how morphine CPP was utilized to understand increased signaling mediated by ERK/CREB in the CeA and not BLA [24]. Thus, we utilized CPP to address long-term effects of cocaine-cue associated neuroplasticity in the BLA-lateral capsula CeA (lcCeA) synaptic pathway.

Cocaine effects on mesolimbic dopaminergic signaling [25]–[35] via modulation of dopamine (DA) transmission are important in cue-induced neuroadaptations. DA projections densely innervate the BLA [36] and basal DA levels stay increased in the BLA and CeA one month after cocaine even without re-exposure to the drug [11]. In addition, autoradiography studies indicate that the BLA-CeA region of the amygdala [37] are among the subregions with the highest density of D1/5R and type 2-like (D2R) receptors [38]. Incidentally, infusing a D1/5R antagonist into the BLA attenuates reinstatement of cocaine seeking behavior [26], suggesting that cue-induced synaptic changes are mediated through D1/5Rs in the BLA.

Long-term potentiation (LTP) is extensively used as a measure of cellular mechanisms underlying synaptic plasticity. In the hippocampus [39] and prefrontal cortex (PFC) [40], LTP is influenced by D1/5Rs. DA gates LTP induction that occurs via suppression of feedforward inhibition from local interneurons in the amygdala [41]. Importantly, effects on LTP mechanisms within the amygdala associated with cocaine-withdrawal, are implicated during the development and maintenance of addictive behavior [42].

In our previous study using locomotor sensitization, we demonstrated that electrically induced LTP is enhanced in the BLA to lcCeA pathway after a 14-day withdrawal from repeated cocaine administration [43]. The enhanced response is blocked by D1/5R antagonists suggesting that endogenous DA plays a role in synaptic plasticity in the amygdala after cocaine treatment. Additionally, we reported that D1/5Rs mediate a corticotrophin releasing factor (CRF)-induced LTP linking stress to cocaine-induced neuronal plasticity in the amygdala during withdrawal [43]. In the present study, we further investigate a role for D1/5Rs and downstream elements in synaptic changes within the BLA-lcCeA pathway of animals subjected to cocaine CPP.

In addition to DRs, both ionotropic and metabotropic glutamate receptors (mGluRs) are involved in cocaine-induced neuroplasticity [44]. mGluRs have been identified as critical for establishing the cue-reinforcing effects of cocaine [45]–[47]. Particularly, hippocampal application of mGluR1 antagonists attenuated context-cue induced reinstatement of cocaine-seeking behavior [48]. Also, a group I mGluR dependent LTP can be recorded in numerous brain areas [49].

A functional relationship between mGluRs and DRs exists in some brain areas. For example, in the PFC, a D1/5R antagonist reduced postsynaptic mGluR5-dependent depolarization evoked by action potential bursts [50]. Similarly, a D1/5R antagonist and group I mGluR antagonists attenuated electrically induced LTP in the core region of the nucleus accumbens (NAcc) [51]. Likewise, D1/5Rs regulated signaling of group I mGluRs in the globus pallidus [52] and oligomers composed of mGluR5 and D2R are found in striatal cells [53] suggesting possible direct interactions between DRs and mGluRs. Recently, we have reported a role for group I mGluRs in the BLA-lcCeA pathway during withdrawal in cocaine CPP expressing animals [54]. In the present study, we investigated the possibility of a functional interaction between D1/5Rs and group I mGluRs in mediating the expression of cocaine CPP. We also investigated phospholipase D (PLD) as an important downstream target for both D1/5R and group I mGluR signaling in the cue–induced conditioned response to cocaine during withdrawal.

PLD was originally discovered as a lipid modifying enzyme that catalyzes the conversion of phosphatidyl choline into choline and phosphatidic acid [55], [56]. However, a number of recent studies on PLD function have implicated an important role for the two known mammalian isoforms, PLD1 and PLD2, in physiological and pathological roles of brain function [55], [57]–[69], including regulation of exocytosis [70], [71], endocytosis [72] and neurotransmitter release [73], all of which are important mechanisms associated with long-term synaptic plasticity.

Agonist activation of mGluRs can signal via PLD [62], [74]–[78]. Specifically, PLD can be activated by excitatory amino acids such as L-cysteine sulfinic acid (L-CSA), an endogenous agonist of PLD-linked mGluRs [75]. PLD-linked mGluRs in rat hippocampus exhibit signaling that is independent of phospholipase C (PLC), adenylyl cyclase, protein kinase C [77], phosphoinositide-specific PLC or inositol (1,4,5) triphosphate signaling [78]. A specific mGluR that signals via PLD was reported as a group I mGluR, possibly a mGluR5 subtype [76], that is exclusively blocked by 2-(2′-carboxy-3′-phenylcyclopropyl)glycine [PCCG-13], a potent selective antagonist of PLD activity [79], [80]. L-CSA blocks while PCCG-13 facilitates a group I mGluR agonist-induced prolongation of epileptiform bursting (another form of synaptic plasticity) [81].

In addition to PLD-linked mGluR studies, there is evidence directly linking DA to PLD activation. D1/5R-mediated Na^+^ current in *Aplysia* neurons is facilitated by PLD activation [65], [82] suggesting that DA transmission is associated with PLD activity downstream. Overexpression of PLD2 in rat substantia nigra causes severe neurodegeneration of DA neurons, a loss of striatal DA, and an associated ipsilateral amphetamine-induced rotational asymmetry suggesting that PLD2 may be pathologically involved in DA release or reuptake [83]. Lastly, PCCG-13 blocks the PLD activation of norepinephrine, a downstream product of DA biosynthesis, in adult rat hippocampus [80]. These observations imply that PLD could be a convergent target that is potentially important in neurotransmission downstream to both dopaminergic and glutamatergic signaling.

Given the link between DR and PLD, mGluR and PLD, the availability of a selective antagonist for the PLD-linked mGluR, and our previous data [43], we focused on DR-mGluR interactions and tested whether in the BLA-lcCeA pathway of cocaine CPP animals: 1) DA induces a long lasting effect on synaptic transmission in slices from cocaine CPP animals; 2) D1/5R agonist-induced synaptic plasticity is dependent on group I mGluRs and the PLD-linked isoform; 3) changes in PLD protein expression are present in amygdala of cocaine CPP animals and whether the pharmacological sensitivity of PLD activity correlates with the D1/5R agonist-induced plasticity including sensitivity to the PLD-linked mGluR antagonist; and 4) inhibiting the PLD-linked mGluR in the amygdala prevents the expression of the cue-conditioned response to cocaine.

## Results

### Robust conditioning to cocaine-cues is measured in animals trained in a counterbalanced CPP paradigm after two weeks withdrawal

**Figure 1:**
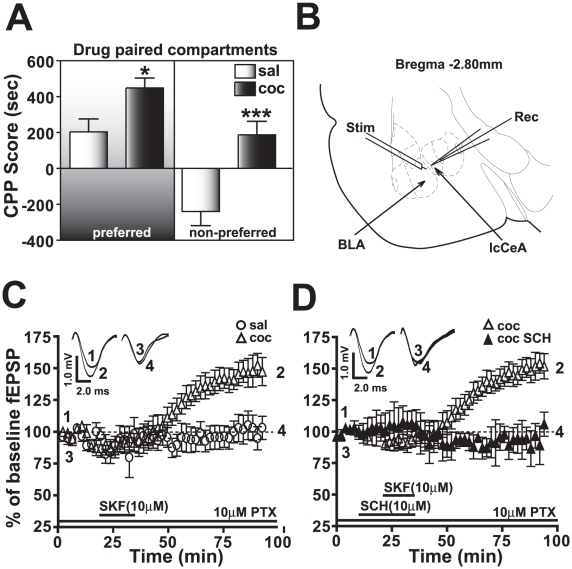
Amygdala slices (B) from animals exhibiting robust cocaine CPP (A) measured 14 days after the last day of CPP training demonstrate a D1/5R agonist-induced LTP in the BLA-lcCeA pathway (C) which is abolished by D1/5R antagonist application (D). A) The cocaine CPP group (black bars) had significantly greater CPP scores than saline-treated animals (white bars) irrespective of whether the drug pairing was on the preferred (saline: 187.1±75.1, cocaine: 448.2±55.7, *p<0.05, n = 34) or the non-preferred side (saline: −239.7±78.5, cocaine: 203.8±71.7, ***p<0.005, n = 34). B) Placement of recording (Rec) and stimulating (Stim) electrodes are indicated in a schematic representation of the slice containing BLA-lcCeA pathway using a rat brain atlas template [127]. C) D1/5R agonist (SKF81297) induces LTP in the BLA-lcCeA pathway in brain slices from animals conditioned to cocaine and tested two weeks after the last CPP training day (clear triangles, 151.4±8.8%, *p<0.05, n = 6). The saline-treated group did not show potentiation (clear circles, 101.6±9.7%, ns, n = 7). Responses are plotted as percent change from the baseline field EPSPs as a function of time. Numbers on the representative traces show the time on the graph at which they were recorded. D) SKF81297-induced LTP in the amygdala from slices of cocaine CPP animals (clear triangles) is completely abolished by the D1/5R antagonist, SCH23390 (filled triangles, 94.5±10.9%, **p<0.01, n = 4). Significance is denoted by increasing number of asterisks (*). For comparison panels C and D use same data graphs and fEPSP traces for the slices from cocaine CPP group superfused with SKF81297.

Two weeks after the last injection, the cocaine CPP group had significantly greater CPP scores than saline-treated animals irrespective of whether the drug pairing was on the preferred side (saline: 187.1±75.1, cocaine: 448.2±55.7, *p<0.05, n = 34) or the non-preferred side (saline: -239.7±78.5, cocaine: 203.8±71.7, ***p<0.005, n = 34, Figure 1A). ‘Preferred’ side indicates the natural preference of the animal for the side with dark floor and dark walls, while ‘non-preferred’ side has white floor and white walls.

The negative CPP score in saline-treated animals suggests that less time was spent on the white side (non-preferred) compared to the black side (preferred). When cocaine injections were associated with the white side, we recorded a positive CPP score indicating that the cues linked with cocaine CPP resulted in the behavioral preference to the normally aversive white-sided environment. Interestingly, associating cocaine with the preferred black side during training also produced enhanced cue-associated response, where the cocaine group spent significantly more time on the black side compared to the saline-treated group. Using these animals, 14 days after the last cocaine CPP training, we analyzed the synaptic changes in the BLA-lcCeA pathway.

### SKF81297 induces LTP in the BLA-lcCeA pathway in the cocaine CPP but not saline-treated group

Since infusing a selective D1/5R antagonist into the BLA attenuated cue-induced reinstatement of cocaine-seeking behavior [26], we analyzed the effects of SKF81297 (10 µM), a selective D1/5R agonist on fEPSPs in the BLA-lcCeA pathway. The fEPSPs in the saline-treated group did not significantly differ from baseline (101.6±9.7%, ns, n = 7) while in the cocaine CPP group, SKF81297 induced a long lasting potentiation of fEPSPs for the duration of the recording (151.4±8.8%, *p<0.05, n = 6, Figure 1C). After one hour washout of SKF81297, fEPSPs in the cocaine conditioned group were significantly greater than in the saline-treated group (***p<0.005) and persisted similar to electrical- and CRF-induced LTP previously recorded in the BLA-lcCeA pathway [43]. This prolonged elevation in fEPSP magnitude also resembled chemically induced LTP described for numerous other drugs [84]–[86]. In addition, SKF81297-induced LTP in the cocaine CPP group was completely blocked (94.5±10.9%, **p<0.01, n = 4, Figure 1D) by a D1/5R antagonist (SCH23390, 10 µM) indicating that SKF81297-induced LTP in the amygdala of the cocaine CPP group was dependent on D1/5Rs. When a higher concentration (25 µM) of SKF81297 was tested, the fEPSP response of saline-treated group (103.0±6.9%, ns, n = 5, data not shown) was not different from that seen with 10 µM SKF81297. Although 25 µM SKF81297 elicited a significant increase in fEPSP magnitude in the cocaine CPP group (135.5±5.9%, *p<0.05, n = 4, data not shown) compared to the saline-treated group at the same concentration, the effect was not significantly different from that of 10 µM concentration (ns). As a result, we used the 10 µM SKF81297 concentration that induced optimal LTP in subsequent experiments.

To test whether synaptic efficacy was altered between the cocaine CPP group, saline-treated group and naïve rats, we measured their input/output responses (I/O). Curves were generated in each slice by measuring lcCeA fEPSPs elicited in response to a series of increasing electrical stimuli applied to the BLA. No significant differences were observed between the groups (see Figure S1), suggesting that synaptic strength did not change.

### SKF81297-induced LTP mimics the DA-induced LTP in the presence of raclopride

**Figure 2:**
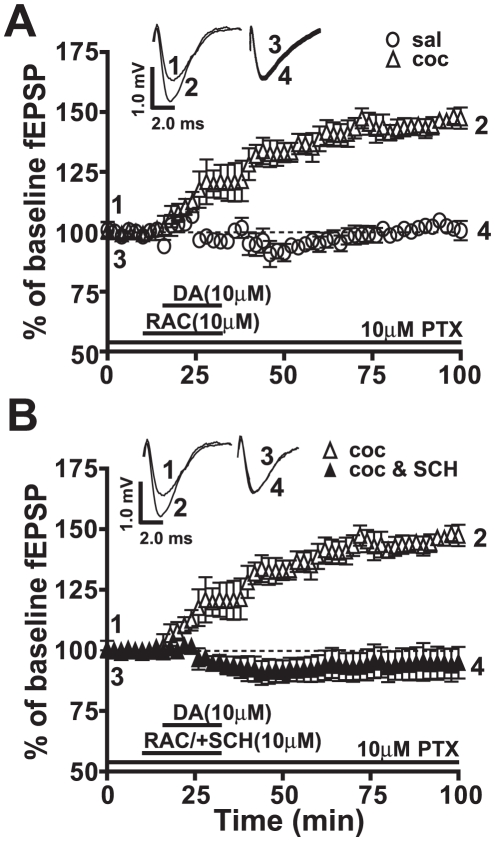
The endogenous neurotransmitter, dopamine (DA), in the presence of raclopride (RAC), a D2R antagonist, induced LTP mediated through D1/5R activation. Responses are plotted as percent change of baseline fEPSPs as a function of time. Numbers on the representative traces show the time on the graph at which they were recorded. A) DA in the presence of RAC (clear triangles, 146.5±3.2%, *p<0.05, n = 5) shows potentiation similar to that recorded with SKF81297 (Figure 1B) in amygdala slices from cocaine CPP animals while the saline-treated group (clear circles, 102.2±2.4%, ns, n = 5) exhibits no LTP. B) DA+RAC-induced LTP is abolished by SCH23390 in the cocaine CPP group (filled triangles, 94.5±4.5%, ns, n = 6). For comparison panels A and B use same data graphs and fEPSP traces for the slices from cocaine CPP group superfused with SKF81297.

To determine if SKF81297 mimics endogenous neurotransmitter-activated D1/5Rs (Figure 2A), we applied exogenous DA (10 µM) in the presence of the D2R receptor antagonist, raclopride (RAC, 10 µM). The DA+RAC-induced LTP recorded in slices from the cocaine CPP group was significantly greater than baseline values (146.5±3.2%, *p<0.05, n = 5) and from the fEPSP values recorded in the saline-treated group (102.2±2.4%, *p<0.05, n = 5). Also, no significant differences were observed between last 10 min fEPSP values of SKF81297- or DA+RAC-induced LTP (ns, n = 5). However, the SKF81297-induced LTP showed a slower onset yet steeper slope before reaching saturation (Figure 1B and 2A).

The ability of DA to induce LTP in the presence of the D2R antagonist indicates that D1/5Rs are the likely receptors mediating the potentiation in the cocaine CPP group. Adding a D1/5R antagonist, SCH23390, completely blocked (94.5±4.5%, ns, n = 6, Figure 2B) the DA+RAC-induced LTP in slices from the cocaine CPP group, confirming D1/5R as the receptor subtype mediating DR-induced LTP.

### GABAergic inhibition is necessary for SKF81297-induced LTP

**Figure 3:**
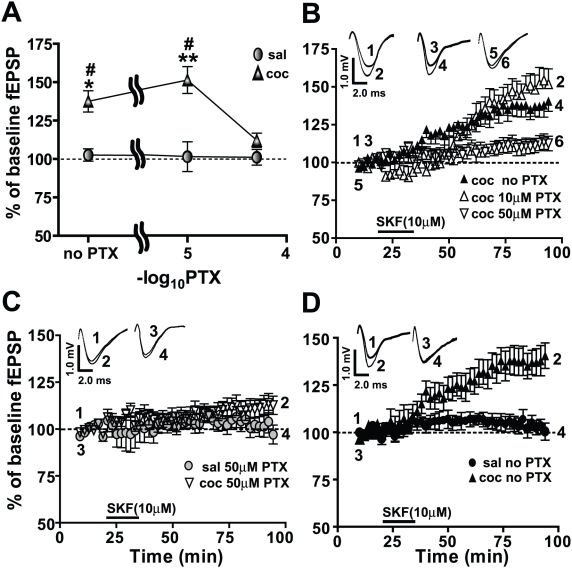
GABAergic inhibition in the BLA-lcCeA synapse is essential for SKF81297-induced LTP in the cocaine CPP group. Field EPSP magnitude is plotted with respect to baseline values as a function of increasing PTX concentrations. Responses are plotted as percent change from the baseline fEPSPs as a function of time. Numbers on the representative traces show the time on the graph at which they were recorded. A) In the cocaine CPP group (filled triangles), SKF81297-induced LTP is lost when GABAergic inhibition is blocked with 50 µM PTX (saline: 101.1±5.1%, cocaine: 112.2±4.6%, ns, n = 7). In the saline-treated groups (filled circles: 102.6±4.0%, 101.6±9.7% and 101.1±5.1%, ns, n = 5–7), the SKF81297-associated fEPSPs do not show a dependence on the extent of GABAergic inhibition. B) SKF81297-induced LTP at different concentrations of PTX in amygdala slices from the cocaine CPP group is plotted as a function of time. LTP in 50 µM PTX (inverted triangles, 112.2±4.6%, n = 7) is inhibited compared to LTP in 10 µM PTX (clear triangles, 151.4±8.8%, n = 6) or LTP in no PTX (filled triangles, 137.5±6.9%, n = 5). C) SKF81297-induced LTP in 50 µM PTX (inverted clear triangles) in the cocaine CPP group is diminished to levels recorded in the saline-treated group (filled circles). D) With GABAergic inhibition intact, SKF81297-induced LTP is significantly increased in the cocaine CPP group (filled triangles) compared to the saline-treated group (filled circles). Panels use same data graphs as in A, B and fEPSP traces in B to illustrate the comparisons. For comparison, all panels use same data graphs and fEPSP traces for the slices from cocaine CPP group superfused with SKF81297 in 10 µM PTX represented in Figures 1 and 2.

Previous studies from this laboratory have routinely utilized 10 µM of the noncompetitive GABA antagonist, PTX, to record fEPSPs in the BLA-lcCeA pathway [43], [54], [87]–[89]. Since DRs are located on γ-aminobutyric acid (GABA) interneurons in the amygdala [90], it is likely that inhibitory transmission could affect the LTP recorded. To examine the relationship between GABAergic inhibition and the SKF81297-induced LTP, we analyzed the dose-dependent effects of PTX (Figure 3). In the cocaine CPP group, SKF81297-induced LTP was abolished in 50 µM PTX (112.2±4.6%, n = 7), compared to LTP in 10 µM PTX (151.4±8.8%, n = 6) or in no PTX (137.5±6.9%, n = 5). The SKF81297-associated fEPSPs in the saline-treated groups were not affected by different levels of GABA inhibition (102.6±4.0%, 101.6±9.7% and 101.1±5.1%, ns, n = 5–7 at 0, 10 and 50 µM PTX, respectively). In contrast, we measured a significant dependence of the SKF81297-induced LTP on the level of GABAergic inhibition in the cocaine conditioned group (Figure 3A). Two-way ANOVA showed a significant effect in the cocaine CPP group (drug) treatment (F_(1,36)_ = 30.04, ****p<0.001), PTX concentration (F_(2,31)_ = 4.48, *p<0.05) and the drug X concentration interaction (F_(2,31)_ = 4.198, *p<0.05).

In 50 µM PTX, fEPSP magnitudes in slices from the saline-treated group were not altered (ns, n = 7, Figure 3C) and were not significantly different than cocaine CPP animals (ns, n = 7, Figure 3C). Thus greater inhibition (50 µM PTX) of GABAergic responses resulted in diminishing the differences in fEPSP magnitudes between cocaine CPP and saline-treated groups measured with 10 µM PTX. In the absence of PTX, fEPSP responses in slices from the saline-treated group were not different from baseline (ns, n = 5, Figure 3D) but SKF81297-induced LTP was significantly different in slices from cocaine CPP animals (*p<0.05, n = 5, Figure 3D). Although LTP measured without PTX and LTP recorded in 10 µM PTX were not significantly different in slices from cocaine CPP animals (ns, n = 5, Figure 3B), we used 10 µM PTX in all subsequent experiments to maximize the signal to noise ratio and to compare these data with our previous studies [43], [54], [87]–[89]. Altogether, these data indicated that intact synaptic inhibition was required for SKF81297-induced LTP in the BLA-lcCeA pathway in the cocaine CPP group.

### PCCG-13, a specific PLD-linked mGluR antagonist, blocks SKF81297-induced LTP

**Figure 4:**
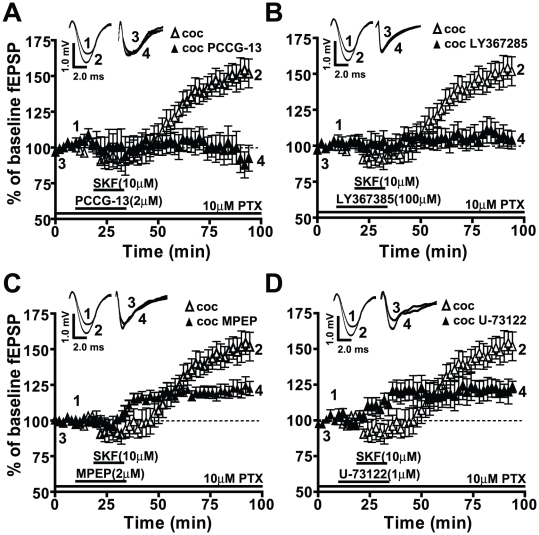
SKF81297-induced LTP in the cocaine CPP group is dependent on the PLD-linked mGluR, mGluR1, and partially dependent on mGluR5 and PLC activity. Responses are plotted as percent change from the baseline fEPSPs as a function of time. Numbers on the representative traces show the time on the graph at which they were recorded. A) SKF81297-induced LTP in the cocaine CPP group (clear triangles, 151.4±8.8%, *p<0.05, n = 6) is completely blocked by the PLD-linked mGluR antagonist (PCCG-13, filled triangles, 95.0±9.2%, n = 6). B) mGluR1 receptor antagonist (LY367385, filled triangles, 106.0±6.7%, n = 6) blocks the SKF81297-induced LTP (clear triangles, *p<0.05, n = 6). C) The mGluR5 antagonist (MPEP, filled triangles, 122.7±5.6%, n = 6) significantly reduces but does not abolish the SKF81297-induced LTP (clear triangles, *p<0.05, n = 6). D) PLC antagonist (U-73122, filled triangles, 128.2±6.1%, n = 6), reduces but does not eliminate the SKF81297-induced LTP (clear triangles, *p<0.05, n = 6). For comparison, panels A and B use same data graphs and fEPSP traces for the slices from cocaine CPP group superfused with SKF81297 as shown in Figures 1, 2 and 3.

The DA system can be linked to PLD [65], [83], [91] and DRs are known to have a functional relationship with group I mGluRs [50], [51], [92]. For these reasons, we examined the possible interaction between DRs and the PLD-linked mGluR by analyzing the effect of a specific antagonist, PCCG-13, which interferes with PLD activity by blocking the PLD-linked mGluR [80]. In the presence of PCCG-13 (2 µM), fEPSP magnitudes in slices from both the saline-treated (SKF81297+PCCG-13: 104.3±8.1%, ns, n = 6, data not shown) and cocaine CPP group (SKF81297+PCCG-13: 95.0±9.2%, ns, n = 8, Figure 4A) were not significantly different from the baseline indicating that the SKF81297-induced LTP in the cocaine CPP group was completely blocked by PCCG-13 (**p<0.01, n = 6, Figure 4A). We also tested the effects of PCCG-13 on the expression of SKF81297-induced LTP, 60 minutes after the washout of the superfused SKF81297. PCCG-13 (97.8±3.1%, n = 4) blocked the expression of SKF81297-induced LTP (150.4±6.9%, ***p<0.005, n = 4) compared to baseline (100.0±3.2%, n = 4, Figure S2).

Additionally, fEPSPs in the presence of PCCG-13, SKF81297, and 50 µM PTX in slices from either the saline-treated group (92.7±9.7%, ns, n = 5, data not shown) or the cocaine CPP group (93.7±10.1%, ns, n = 5, data not shown) were not significantly different from the baseline. These data suggest that the SKF81297-induced LTP in slices from cocaine CPP animals may be dependent on mGluR modulation of PLD.

To examine further the mGluR subtype linked to PLD, we tested the effect of LY367385 (100 µM), a competitive mGluR1 antagonist. While fEPSP magnitudes were not significantly different from baseline in slices from the saline-treated group (109.3±8.4%, ns, n = 6, data not shown), LTP in the presence of LY367385 was blocked in slices from the cocaine CPP group (SKF81297+LY367385: 106.0±6.7%, ****p<0.001, n = 6, Figure 4B). MPEP (10 µM), the competitive mGluR5 antagonist, significantly reduced the SKF81297-induced LTP in slices from the cocaine CPP group (SKF81297+MPEP: 122.7±5.6%, **p<0.01, n = 6, Figure 4C), indicating that mGluR5 activation also contributed to the SKF81297-induced LTP. However the remaining LTP was significantly greater than baseline (*p<0.05) and from that in LY367385 (*p<0.05), indicating that MPEP only diminished, while LY367385 completely blocked the LTP. MPEP induced no significant changes in fEPSP responses in the saline-treated group (107.8±3.4%, ns, n = 9, data not shown) compared to baseline (100.0±3.5%, n = 9, data not shown). This suggests that both group I mGluRs (mGluR1 and mGluR5) can mediate the SKF81297-induced LTP in the cocaine CPP group but only the mGluR1 antagonist mimicked the effect of PCCG-13.

Since the link between group I mGluRs and signaling via the phospholipase C (PLC) in the brain is well established [93]–[95], we tested the effect of a PLC inhibitor, U-73122 (1 µM), on the SKF81297-induced LTP. While U-73122 attenuated fEPSP response in the cocaine CPP group (SKF81297+U-73122: 128.2±6.1%, ***p<0.005, n = 6, Figure 4D), LTP magnitude was still significantly greater than either baseline (*p<0.05) or fEPSP after PCCG-13 application (*p<0.05). U-73122 did not produce any significant change in the fEPSP response in the saline-treated group (110.3±5.3%, ns, n = 5, data not shown) compared to baseline (100.0±2.4%, n = 5, data not shown). These data suggest that the SKF81297-induced LTP may involve PLC and PLD signaling. On the other hand, it was recently reported that U-73122 can also inhibit cardiac PLD activity [96] suggesting that PLC mediation of D1/5R agonist-induced LTP may be due to its effect on PLD.

### Expression of amygdala PLD and not DR or mGluR protein levels is elevated in cocaine CPP group

**Figure 5:**
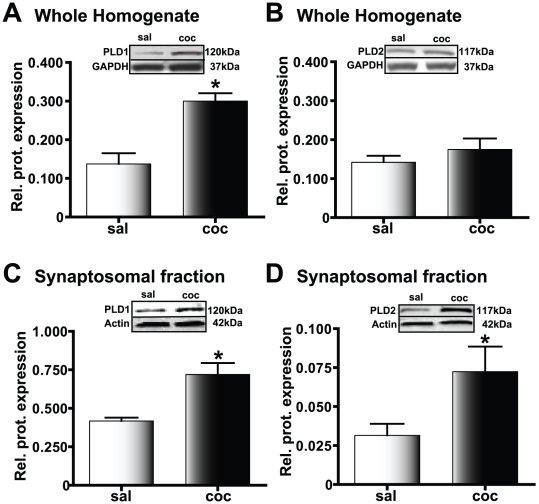
PLD levels in the amygdala are increased in cocaine CPP animals. Protein expression relative to the loading control is plotted along the Y-axis. Representative immunoblots are shown in the panels above each graph; *p<0.05 compared to the corresponding saline-treated control. A) PLD1 levels in the whole amygdala homogenate are significantly increased in cocaine CPP animals (black bars) compared to the saline-treated group (white bars). B) PLD2 levels in the whole amygdala homogenate are not increased in the cocaine CPP group (black bars) compared to the saline-treated group (white bars). C) In the amygdala crude synaptosomal fraction, PLD1 protein levels are increased in the cocaine CPP group (black bars) suggesting that there is increased synaptosomal membrane incorporation of PLD1 in this experimental group compared to the saline-treated group (white bars). D) Similar to PLD1, amygdala crude synaptosomal levels of PLD2 show an increase in the cocaine CPP group (black bars). However, such increased expression is observed despite a lack of increase in the whole homogenate levels, suggesting that recruitment from the existing pool of PLD2 to the synaptosomal membrane is increased in the cocaine CPP group.

When we studied the protein expression levels using Western blot analysis, PLD1 expression was significantly increased in whole cell homogenate of amygdala obtained from cocaine CPP animals compared to the saline-treated group (**p<0.01, n = 4, Figure 5A). However, no significant difference in PLD2 protein expression was detected (ns, n = 4, Figure 5B) suggesting that PLD1, not PLD2, could be the PLD subtype linked to the mGluR mediating SKF81297-induced LTP. When we further tested crude synaptosomal levels in cocaine CPP group, both PLD1 (*p<0.05, n = 3, Figure 5C) and PLD2 (*p<0.05, n = 3, Figure 5D) expression were significantly increased, suggesting that both isoforms of amygdala PLD are affected in the conditioned response to cocaine.

Western blot analyses performed on proteins obtained from either whole amygdala homogenate or crude synaptosomal fractions failed to show a significant difference in protein expression levels for either DRs (D1R or D5R) or mGluRs (mGluR1 and mGluR5) in cocaine CPP and saline-treated groups (data not shown). This suggests that the mechanism contributing to the role of DRs and mGluRs in SKF81297-induced LTP in the cocaine CPP group does not involve changes in overall receptor levels.

### PLD1 and PLD2 associate with mGluR1 and mGluR5 in the amygdala of cocaine CPP animals

**Figure 6:**
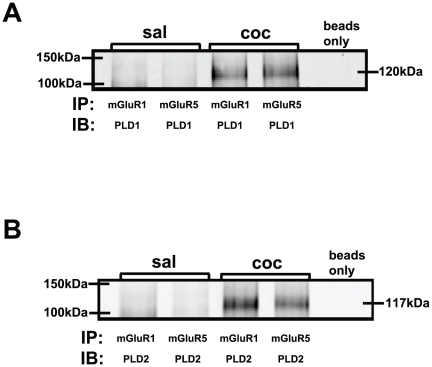
Amygdala protein subjected to co-immunoprecipitation (IP) with mGluR1, mGluR5 and subsequently immunoblotted (IB) with PLD antibodies show association between PLD1/2 and mGluRs1/5 in the cocaine CPP group. A) PLD1 and B) PLD2 were detected only in the amygdala of cocaine CPP group (coc) but not saline-treated group (sal).

Since the SKF81297-induced LTP was blocked with antagonists for both PLD-linked mGluR and group I mGluRs, we examined whether PLD is associated with mGluR1 or mGluR5 using co-immunoprecipitation assays. Both PLD1 (Figure 6A) and PLD2 (Figure 6B) were immunoprecipitated by mGluR1 and mGluR5 antibodies in the amygdala extracts from cocaine CPP animals and not in the saline-treated group.

Reciprocal co-immunoprecipitations using PLD1 or PLD2 and co-immunoprecipitations with D1R or D5R antibodies could not be analyzed since the co-immunoprecipitations showed only immunoglobulin bands. It is well established that not all antibodies employed with Western blotting can be utilized for immunoprecipitations. This incompatibility is attributed to inaccessibility of epitopes when the protein is in its native conformation during immunoprecipitation [97].

### PLD activity in BLA/CeA containing slices from cocaine CPP, but not saline-treated animals, is altered in response to treatment by agonists and antagonists of D1/5R and mGluRs

**Figure 7:**
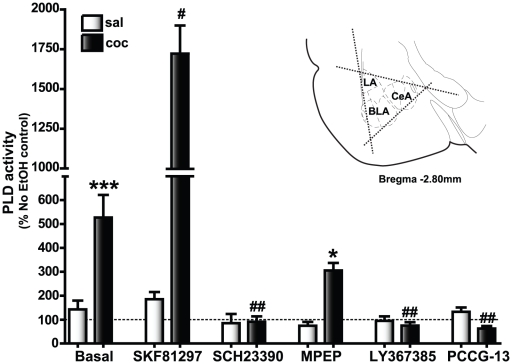
Basal PLD activity is strongly stimulated by the D1/5R agonist and blocked by the D1/5R, mGluR5, mGluR1, and the PLD-linked mGluR antagonists in the amygdala of cocaine CPP animals. The dotted line indicates PLD activity associated with control slices (no EtOH added) which was determined for each animal and used to calculate the change in PLD activity levels with EtOH and/or drug application. Basal levels represent the increase in PLD activity observed in the EtOH-treated slices compared to the no EtOH controls; *p<0.05 compared to the corresponding saline control and ^#^ *p*<0.05 compared to the cocaine CPP group basal PLD activity. Basal PLD activity was significantly increased (***p<0.001, n = 50) in the cocaine CPP group (dark bars, 527.3±94.3) compared to the saline-treated group (white bars, 142.6±36.9). SKF81297, the D1/5R agonist, application increased the basal levels in the cocaine CPP group significantly (1722.0±176.9, n = 12, ^#^ *p*<0.05) compared to the basal PLD activity observed with EtOH treatment alone in the same experimental group. The D1/5R antagonist, SCH23390, completely blocked basal PLD activity (91.2±21.9, n = 12, ^##^ *p*<0.01) in the cocaine CPP group. A similar reduction in PEtOH levels was observed with application of either the PLD-linked mGluR antagonist, PCCG-13 (62.9±10.6, n = 7, ^##^ *p*<0.01) or the mGluR1 antagonist, LY367385 (75.0±13.9, n = 12, ^##^ *p*<0.01), while the mGluR5 antagonist, MPEP, did not decrease basal PLD activity (305.7±31.5, n = 7, ns) within the cocaine CPP group but were significantly increased compared to (*p<0.05) the saline treated group. Applications of SKF81297 (184.9±30.5, n = 12), SCH23390 (84.9±38.9, n = 12), MPEP (74.2±16.3, n = 7), LY367385 (94.7±18.9, n = 12) and PCCG-13 (132.5±18.4, n = 7) did not significantly alter the PEtOH levels in the saline-treated group compared to the basal activity levels. Inset is a depiction of the triangular excision performed to isolate amygdala (bilaterally for each animal, each slice) containing the basolateral (BLA), the central (CeA) and the lateral (LA) subregions from three serial coronal slices (350 µm) beginning −2.30 mm to −2.80 mm from Bregma [127].

An enzymatic activity assay [98] was used to determine whether the increase in amygdala PLD protein expression in cocaine CPP group reflected an increase in PLD activity (Figure 7). Baseline PLD activity was increased in the BLA/CeA containing slices from the cocaine CPP group (527.3±94.3, n = 50) compared to the saline-treated group (142.6±36.9, ****p<0.001, n = 50). We also tested the effect of D1/5R agonist (SKF81297), D1/5R antagonist (SCH23390), mGluR1 antagonist (LY367385), mGluR5 antagonist (MPEP), and specific PLD-linked mGluR antagonist (PCCG-13) on baseline PLD activity. Applications of SKF81297 (184.9±30.5, n = 12), SCH23390 (84.9±38.9, n = 12), LY367385 (94.7±18.9, n = 12), MPEP (74.2±16.3, n = 7) and PCCG-13 (132.5±18.4, n = 7) did not significantly alter the phosphatidyl ethanol (PEtOH) levels in the saline-treated group compared to the basal activity levels. The D1/5R agonist, SKF81297, strongly stimulated the enhanced basal PLD activity in cocaine CPP group (1722.0±176.9, *p<0.05, n = 12) and the D1/5R antagonist (SCH23390) decreased basal PLD activity in the cocaine CPP group (91.2±21.9, **p<0.01, n = 12). Addition of PCCG-13 (62.9±10.6, n = 7) and LY367385 (75.0±13.9, n = 12) also significantly reduced basal PLD activity in BLA/CeA slices from cocaine CPP animals (**p<0.01, n = 7) but not the saline-treated group suggesting that the increased basal PLD activity in the cocaine group was mediated through D1/5Rs, mGluR1 and the PLD-linked mGluR. On the other hand, the mGluR5 antagonist (MPEP) application reduced but did not block basal PLD activity (305.7±31.5, ns, n = 12) in the cocaine CPP group suggesting that mGluR5 associated changes in the SKF81297-induced LTP in cocaine CPP animals may be mediated through mechanisms other than those associated with an increase in PLD activity. Overall, these data indicated that the pharmacological sensitivity of basal and stimulated PLD activity correlated with that of the SKF81297-induced LTP suggesting that a PLD-linked mGluR mediates the D1/5R agonist-induced LTP in the amygdala of cocaine CPP animals.

### Blocking the PLD-linked mGluR inhibits the expression of CPP after two weeks withdrawal in the cocaine CPP group

To test whether mGluR-linked PLD activation in the amygdala was important for cue-induced behavioral response to cocaine, we studied the effect of PCCG-13 on the expression of CPP after two weeks withdrawal. Bilateral cannulae were surgically implanted into the BLA to permit direct infusion of PCCG-13 as described earlier. Animals were trained for cocaine CPP on the non-preferred side to increase the signal-to-noise ratio.

**Figure 8:**
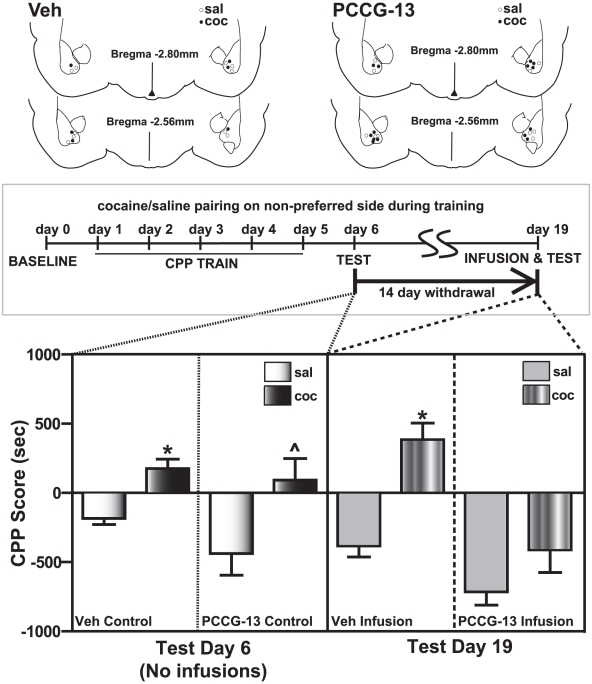
Expression of cocaine CPP was blocked by bilateral infusions of the PLD-linked mGluR antagonist into the amygdala of cocaine CPP group 14 days after the last CPP training. Infusions of the vehicle into the cocaine CPP animals (striped bars, 383.5±121.0, n = 3, Veh infusion panel) on test day 19 do not diminish cocaine CPP (black bars, 176.1±56.3, n = 3, Veh control panel) measured on test day 6. No significant changes were observed in the behavioral response of the saline-treated group between day 6 (white bars, −170.2±36.2, n = 5, Veh control panel) and day 19 (grey bars, −369.2±71.3, n = 5, Veh infusion panel), suggesting that the vehicle infusions alone had no effect. PCCG-13 infusion 15 minutes prior to CPP testing results in a marked loss of preference for the drug-associated side (black bars, cocaine day 6: 92.4±145.9, striped bars, cocaine day 19: −397.1±155.6, *p<0.05, n = 7, PCCG-13 control and PCCG-13 infusion panels respectively) in the cocaine CPP animals, while no significant change in the behavioral response is observed in saline-treated group (white bars, saline day 6: −423.5±149.7, grey bars, saline day 19: −700.8±143.2, ns, n = 5, PCCG-13 control and PCCG-13 infusion panels respectively). *p<0.05 compared to the corresponding vehicle control (saline) and by ∧*p*<0.05 compared to the corresponding vehicle control (PCCG-13). Cannulae placement for the animals is depicted (above the graph) for both the vehicle (Veh) and drug (PCCG-13). These sites were mapped to BLA on rat brain atlas [127] templates. A schematic representation of the experimental protocol utilized for CPP training along with the two days of CPP testing is illustrated above the corresponding data in the graph below.

PCCG-13 infusion produced a significant difference in the behavior of the cocaine CPP animals (Figure 8). CPP for the drug-paired, non-preferred side in the cocaine group observed on day 6 (one day after last CPP training, 92.4±145.9, n = 7) was not observed after PCCG-13 infusions in the same animals on day 19 (two weeks after last CPP training, −397.1±155.6, ns, n = 7). However, animals receiving PCCG-13 infusions in the saline-treated group did not register a significant difference in the CPP score on day 19 (−700.8±143.2, n = 5) compared to day 6 (−423.5±149.7, n = 5). These data suggested that the PLD-linked mGluR is important for mediating long-term synaptic plasticity in the amygdala associated with cocaine conditioned responses.

Two sets of controls were used in this study. (1) Since BLA is important for acquisition of conditioned responses, we tested animals that were implanted with cannulae and trained for CPP similar to the experimental group, but not given infusions. A robust CPP was observed in the cocaine group, both on day 6 (saline: −87.8±93.4, n = 8, cocaine: 368.8±96.5, n = 9, **p<0.01) and on day 19 (saline: −186.5±126.4, n = 8, cocaine: 296.9±127.8, n = 9, *p<0.05) compared to the saline group, suggesting that animals having cannulae placement in the BLA did not show deficits in CPP acquisition. (2) To control for infusion related effects, we tested animals that were implanted with cannulae and trained for CPP similar to the experimental group, but given the same volume of vehicle infusions before test on day 19 (Figure 8). In this case, CPP was present on both day 6 (176.1±56.3, n = 3) and day 19 (383.5±121.0, n = 3) in the cocaine CPP group compared to saline-treated animals (day 6: −170.2±36.2, *p<0.05; day 19:−369.2±71.3, *p<0.05, n = 5), suggesting that neither the infusion procedures nor the vehicle used affected CPP expression.

## Discussion

The main findings from our present study are: (1) a D1/5R agonist induces LTP in the rat BLA-lcCeA synaptic pathway in the cocaine CPP but not the saline-treated group; (2) the D1/5R agonist-induced LTP is dependent on the PLD-linked mGluR, and mGluR1; (3) amygdala PLD1 and PLD2 (but not DR or mGluR) protein expression are increased in crude synaptosomal fractions from the cocaine CPP group; (4) basal PLD activity is increased in the cocaine CPP group, further stimulated by D1/5R agonist and inhibited by antagonists of mGluR1 and PLD-linked mGluR; (5) PLD1 and PLD2 association with mGluR1 and mGluR5 is only observed in the cocaine CPP group; and (6) the PLD-linked mGluR antagonist blocks the expression of cocaine CPP.

### A D1/5R agonist induced LTP is recorded in the BLA-lcCeA pathway in brain slices from cocaine CPP group after two weeks withdrawal

In our previous study [43], we reported that D1/5R antagonist blocked electrically induced LTP while both D1/5R and D2R antagonists blocked CRF-induced LTP during cocaine withdrawal. Since D1/5Rs were common to both types of LTP, we now focused on their direct activation and potential dependence on mGluRs after 14 days of withdrawal from cocaine CPP. We observed a D1/5R agonist (SKF81297) associated LTP that occurred in the BLA-lcCeA pathway only in the cocaine CPP group which could be completely abolished by a D1/5R antagonist (SCH23390). This is similar to earlier studies where synaptic potentials in the PFC are facilitated by DA after cocaine [99]. DA can facilitate LTP induction in hippocampal neurons by increasing a timing-dependent LTP window and permitting normally ineffective weak stimuli, with fewer spikes, to generate significant LTP [100]. We propose an analogous mechanism where dopaminergic signaling via group I mGluR and PLD transforms the SKF81297 fEPSP response in the saline-treated group into LTP in the cocaine-conditioned group that persists in the amygdala slices long after cessation of drug-intake.

DA, the endogenous ligand, in the presence of a selective D2R antagonist (RAC), also generated LTP similar to the SKF81297-induced LTP. This suggests that DA activation of D1/5Rs, not D2Rs, is important for the LTP observed. Activation by DA is immediate while a 20 minute delay is observed in the SKF81297-induced LTP. This could very well be attributed to the chemistry of SKF81297, a benzazepine that has a functionally mobile phenyl substituent which influences its efficacy in binding and transducing its actions [101], [102]. In studies comparing the effect of D1/5R partial and full agonists on cAMP production and D1R internalization [103], a different rate was attributed to distinct binding characteristics associated with D1/5R and G-protein interface. This suggests that the delayed SKF81297-induced LTP recorded in the present study could be related to agonist structure. Besides, SKF81297 application initially depressed fEPSPs before inducing LTP (Figure 4) whereas DA/RAC-induced LTP had a fast time course and no initial depression of fEPSPs. Interestingly, MPEP and a PLC antagonist (U-73122) blocked the SKF81297 delay resulting in faster onset but depressed LTP magnitude suggesting that the initial depression may be due to activation of a SKF81297-induced long-term depression (LTD), one that is dependent on mGluR5 and PLC but obscured by a larger SKF81297-induced LTP. This proposed mechanism for the depression induced by SKF81297 is supported by evidence showing that group I mGluR-dependent LTD is recorded in many brain areas and ascribed to multiple mechanisms (see [104] for review) and that DR and mGluR agonist together can induce LTD in the PFC [105].

### DRs and GABAergic synapses in the BLA-lcCeA pathway

DA modulation of GABAergic function occurs throughout the BLA-lcCeA pathway [106]. While D1R activation increases excitability through direct stimulation of BLA projection neurons, it also augments the inherent excitability of local BLA interneurons and therefore results in increased inhibition to projection neurons [107]–[109]. In contrast to local BLA interneurons, D1/5R activation hyperpolarizes GABAergic medial paracapsular (intercalated) cells (MPCs), which gate feed-forward inhibition from the BLA to the CeA [110] resulting in increased transmission to the CeA [90], [106]. During withdrawal, GABAergic transmission is reduced in midbrain neurons, which results in electrically-induced LTP [111]. Such reductions in GABAergic transmission could occur in the BLA-lcCeA and influence the D1/5R signaling. Thus, we tested whether changing the level of GABA inhibition would affect the D1/5R agonist-induced LTP. We found that the SKF81297-induced LTP was blocked in 50 µM PTX in the cocaine CPP group. In previous studies, we observed that GABA synaptic transmission in the BLA-lcCeA pathway is blocked with 50 µM PTX where spontaneous GABAergic miniature inhibitory synaptic currents are reduced in frequency and amplitude indicating a reduced GABAergic tone onto lcCeA neurons in amygdala slices from cocaine CPP animals [54]. Thus, DA inhibition of MPCs which reduces GABAergic inhibition on downstream CeA neurons [90] coupled with reduced GABAergic tone onto lcCeA neurons [54] may facilitate the development of LTP in the cocaine CPP group.

### SKF81297-induced LTP is mediated through mGluR1 and PLD-linked mGluR in the cocaine CPP group

In the cocaine conditioned group, antagonists for group I mGluRs blocked the D1/5R agonist-induced LTP in the BLA-lcCeA pathway implicating increased mGluR and/or DR expression as a potential mechanism for development of the SKF81297-induced LTP during cocaine withdrawal. However, increases in protein expression of mGluRs or DRs were not measured in the amygdala of cocaine CPP animals. These results are similar to studies after acute amphetamine treatment which showed increased activity of another signaling partner of DRs, phosphoinositide-3-kinase, that was not accompanied by increase in DR levels [112]. Also, in self-administration studies, D1/5R levels were not different from control levels in the limbic brain regions one week after the last cocaine training [113]. These observations suggest that the SKF81297-induced LTP in the cocaine CPP that we observed could occur by mechanisms other than increased D1/5R levels. For example, D1/5Rs work cooperatively with both mGluR1 and mGluR5 in the NAc to mediate electrically induced LTP [51] while in the PFC, co-activation of DRs and a group I mGluR agonist can together induce a chemical LTD [105]. Also, a group I mGluR antagonist blocked locomotor behavior induced by D1/5R agonists in the NAc [114]. Finally, the presence of oligomers of DRs and mGluRs in living cells [53] provides support for the hypothesis that DR/mGluR heteromers could underlie D1/5R agonist induced LTP in the present study.

With hetero-oligomer formation, non-functional receptors can become operative by associating, while agonists can trigger functional receptors to dimerize to increase efficacy [115]. mGluR5 can dimerize via disulfide bonds in the extracellular N-terminal domain [116] and formation of D1/5R and D2R heteromers activates calcium–calmodulin kinase (Ca^+2^-CaMKII) signaling pathway [117]. Also, disruption of group I mGluR function in the globus pallidus, important for synaptic plasticity, can be restored and regulated by D1/5R and D2R activation [52]. In our studies, the D1/5R agonist strongly stimulated amygdala PLD activity in the cocaine CPP group indicating a biochemical link between D1/5Rs and PLD. Also, PLD-group I mGluRs co-immunoprecipitation studies indicate a direct physical link between mGluRs and PLD in amygdala. Thus, we propose that a co-operative interaction between mGluR and DR perhaps via formation of a heterodimer may lead to an increase in membrane binding of PLD1/PLD2 and underlie the SKF81297-induced LTP recorded in the amygdala of cocaine CPP animals.

### Amygdala PLD activity and expression are increased in cocaine CPP animals

Despite no change in the protein levels of both DRs and mGluRs in the amygdala, PLD1 and PLD2 crude synaptosomal expression were elevated 2–2.5 fold indicating a potential increase in interaction of both PLD1 and PLD2 with the existing D1/5Rs and group I mGluRs in the cocaine CPP group. The elevated PLD expression was accompanied by increased PLD activity. Basal amygdala PLD activity increased 3.9 fold in the cocaine CPP group. In addition, PLD activity in the presence of the D1/5R agonist was increased 9.3 fold over the agonist-stimulated saline-treated group. Basal amygdala PLD activity was blocked completely by D1/5R, PLD-linked mGluR, and mGluR1 antagonists in amygdala slices from cocaine CPP group but only diminished by an mGluR5 antagonist to levels still significantly higher than corresponding saline-treated group. Similar agonist-like effects of mGluR antagonists on PLD activity have been reported previously [74]–[78]. These findings of agonist and antagonist effects on basal PLD activity directly correlate with those of D1/5R-agonist induced LTP suggesting that increased PLD activity could be a mechanism associated with SKF81297-induced LTP in the cocaine CPP group. It has been reported that an elevated PLD activity, with no change in PLC expression, can generate a stable signaling pathway [66], perhaps more suitable for the SKF81297-induced LTP observed.

### Cocaine CPP associated long-term memory is blocked by the PLD-linked mGluR antagonist

In the rat preclinical model, cocaine conditioned behaviors are blocked by infusion of a D1/5R antagonist into the amygdala [26] and by group I mGluR antagonists in the hippocampus [48] suggesting an important contribution by both receptors to cue-induced cocaine responses. In the present study using PCCG-13, we clearly demonstrate that amygdala PLD-linked mGluR signaling is important for the expression of both cocaine CPP and SKF81297-induced LTP. Thus, PLD-mediated signaling may be a necessary step in activating events downstream from mGluRs and DRs in amygdala synaptic plasticity. Rat brain PLD1/PLD2 expression that occurs throughout development and stabilizes during adulthood [118], [119], could govern physiological processes of neurite outgrowth [64], [120]–[124] and neurotransmitter release [73], thus regulating cellular synaptic plasticity that affects long-term memory mechanisms. Indeed, a recent study of synaptic dysfunction in a mouse model of Alzheimer's disease implicated PLD as an important element that can regulate underlying memory deficits [68]. Thus, further investigation of PLD interactions may be key to better understanding molecular correlates of relapse to cocaine providing a framework for developing therapeutic interventions that successfully target addiction.

## Materials and Methods

### Animals

All animal procedures were carried out in accordance with the Guide for the Care and Use of Laboratory Animals as adopted and promulgated by the National Institutes of Health (NIH) and approved (Approval ID: 8907176) by the Institutional Animal Care and Use Committee (IACUC) at the University of Texas Medical Branch at Galveston (UTMB). Male Sprague-Dawley albino rats (Harlan, Houston, TX, USA), age 3–4 weeks and weighing approximately 45 grams during arrival, were used as subjects. After 3 days acclimation, animals were randomly divided into cocaine and saline groups and housed in a temperature-controlled room at 22–24°C with a 12 hr light/dark cycle and fed a standard laboratory chow diet and water *ad libitum.*

### Conditioned place preference (CPP) *apparatus*

Four acrylic animal chambers (16’’ X 16’’ X 12’’) were contained inside of sound and light attenuating environmental control boxes (Accuscan Instruments, Inc, Columbus, OH). The chamber was subdivided into two distinct compartments, one with white floors and walls associated with a textured floorboard (raised Plexiglas ridges), the other with black floors and walls on a smooth Plexiglas floorboard. The light intensity in the chambers was maintained at 320 lumens. On baseline and testing days, the animal was placed in a removable holding chamber (6’’ X 3’’ X 6’’) that was inserted centrally between the black and the white compartments, and then lifted, to allow the animal free access to both sides. On conditioning days, a centrally inserted removable 12’’ acrylic single pane wall restricted the animal to one compartment. Activity and time spent on each side were measured using the VersaMax activity monitor system (Accuscan Instruments, Inc).

### Experimental design

The counterbalanced CPP behavioral paradigm included three sessions: baseline, conditioning and testing. The baseline and testing sessions were performed only once, while the conditioning sessions occurred over 5 days. For the baseline session (performed 24 hr prior to the first injection), each animal was allowed to freely roam the activity box for 30 min in order to test the animal's preference for one side over the other (i.e., a biased design). Animals were then randomly assigned to be in the cocaine-treated experimental or saline-treated control groups. Conditioning sessions were 30 min long during which saline or cocaine injection delivered intraperitoneally (i.p.) was paired with either the black or the white side. In the morning, all animals received saline injections (1 ml/kg of 0.9% saline solution). During each experiment, equal number of animals were placed in the different chambers (either the black or the white side). In the afternoon, the control animals received saline, while the experimental animals were administered cocaine. Sessions were counterbalanced. The animals placed on the white side in the morning were placed on the black side in the afternoon and those on the black side in the morning were placed on the white side in the afternoon. In the afternoon, all animals also received sound and light cues; for the first 5 min of the session (once per second) the animals received a tone (70 db white noise), simultaneously with light (320 lumens on/off per 15 sec). Since the amygdala, in addition to cues mediated by context, also processes cues to light and sound [125], addition of these cues improved the CPP response in our experiments. During the 30 min test session on day 6 (24 hr after the last training session), animals were allowed to roam freely between the two chambers (as in baseline). The testing session also included the light and sound cues that were previously paired with saline or cocaine injections in the afternoon training sessions. The amount of time per side of the chamber was determined and reported as a CPP score [126]: - the time spent on the drug-paired side during test day 6 or 19 minus the time spent on the same side during baseline. If a population of animals spent more time on the drug-paired side during the test sessions than during the baseline session, the CPP score would be positive, suggesting that a conditioned place preference had developed during the training period. Behavior was analyzed using one-way or two-way repeated measures ANOVA followed by paired or unpaired t-test when significance was reached.

### Surgery

Post-acclimation to arrival, rats were anesthetized with i.p. injections of ketamine (90 mg/kg) plus xylazine (10 mg/kg). Based on animal size, age adjustments were made to the co-ordinates to guide the cannulae into the basolateral amygdala (BLA) for infusions. Specifically, bilateral cannulae were stereotaxically placed in the BLA (anterioposterior −2.64 mm from bregma, lateral +/−4.9 mm and dorsoventral −6.3 mm [127]), according to previously described procedures [128]. Animals were allowed 5 days to recover, and subsequently subjected to CPP conditioning for the next 5 days. On day 6 (24 hr post training), the animals were tested for CPP and then subjected to withdrawal in their home cages for 14 days. Following withdrawal, the animals were bilaterally infused with 0.5 µl of either PCCG-13 (8.5 µM) in vehicle (0.0134% beta cyclo-dextrin, BCD, in 0.9% saline) or vehicle alone over a period of 20 minutes and allowed to remain in their home cage for 15 min prior to CPP testing. Twenty four hours after the CPP test, animals were infused with 0.5 µl of methylene blue dye in dimethyl sulfoxide (DMSO), sacrificed and brain slices prepared to verify cannulae placement.

### Slice Preparation

Coronal brain slices were prepared after 14 days withdrawal as described previously [43]. No anesthetics were used prior to decapitation to avoid their influence on neuronal plasticity. Serial coronal slices (500 µm), containing the BLA-lcCeA, were cut (approximately 2.3–2.8 mm from Bregma) [127]. Initially, slices were bathed in oxygenated, modified artificial cerebrospinal fluid (ACSF) solution (in mM): NaCl (119), KCl (3.0), NaH_2_PO_4_ (1.2), MgSO_4_ (1.2), CaCl_2_ (2.5), NaHCO_3_ (25) and glucose (11.5) at room temperature (RT) for 1 hr. They were then submerged in a chamber (1.0 mL, 2.5 mL/min) and held at 30±1°C for another hour before recording. A constant pH (7.4) was maintained by continuous superfusion (2 mL/min) with oxygenated [95% oxygen/5% carbon dioxide (carbogen)] ACSF.

### Electrophysiology

Field excitatory postsynaptic potentials (fEPSPs) were recorded with tungsten electrodes (2–5 MΩ) in coronal brain slices as described previously [43]. Briefly, fEPSPs were evoked by stimulating fibers in the BLA using 150 µs pulses of varying intensity (3–15 V) applied at 0.05 Hz through concentric electrodes (50 kΩ). All experiments were performed in the presence of 10 µM picrotoxin (PTX) in ACSF except where noted. Initially, fEPSP magnitude was adjusted to 30% of maximum response and baseline values recorded for 10 min. For experiments where agonists were superfused, antagonists were added to the ACSF for 10 min prior to addition of the agonist and continued throughout the 15 min agonist superfusion. Thereafter, fEPSPs evoked at a frequency of 0.05 Hz were recorded for 1 hr. Drug-induced changes in fEPSP slopes during drug application, during agonist treatment, and during the last 10 min of the experiment, were calculated and normalized to baseline values.

### Western Blotting

#### Amygdala tissue preparation

After withdrawal, rats were decapitated and the brain was sliced to obtain the amygdala. As described previously [43], the appropriate amygdala subregions were isolated and homogenized with lysis buffer (Mammalian Cellytic lysis buffer; Sigma, St. Louis, MO, USA) containing protease inhibitors (complete mini EDTA-free protease cocktail tablet; Pierce Biotechnologies, Rockford, IL, USA) to obtain amygdala homogenate. The cell membrane fraction was obtained by homogenizing isolated amygdala subregions with lysis buffer containing (in mM): sucrose (320), Tris–HCl (25), EGTA (2), EDTA (2) and protease inhibitors and adjusted to pH 7.4. Individual samples were then centrifuged at 1090 g for 15 min at 4°C and the supernatant was collected into ultra-clear tubes and centrifuged (Beckman Coulter, Inc., Fullerton, CA, USA) twice at 15,000 g for 20 min. The pellet fraction was dissolved in a minimum volume of lysis buffer with 1% Triton-X 100. Protein quantification was performed using the BCA protein assay (Pierce Biotechnologies). Aliquots of 50 µg protein samples were stored at −80°C until further use. Each sample represented one animal.

#### Immunoblotting

A solution of 2X sample buffer with 1 mM dithiothreitol (DTT) was added to the samples and then placed in water bath at 37°C for 60 min followed by 5 min cooling on ice, unless otherwise indicated. Samples (50 µg per lane) were separated on a 10% acrylamide gel by SDS-PAGE and transferred to a nitrocellulose membrane overnight in a cold room. The membranes were then blocked with LI-COR (LI-COR Biosciences, Lincoln, NE, USA) blocking buffer for at least 1 hr at RT or overnight at 4°C and then incubated overnight in primary antibodies (diluted in LI-COR blocking buffer). After removal of the antibody, the blot was washed with phosphate-buffered saline (PBS) [(in mM) NaCl (137), KCl (2.7), Na_2_HPO_4_ (100), KH_2_PO_4_ (2)] containing 0.05% Tween 20 (PBST). All further steps were carried out in the dark to prevent the loss of sensitivity of the infrared dye secondary antibodies. The secondary antibodies (diluted in LI-COR blocking buffer) were applied for 1 hr at RT. The blots were scanned directly by the Odyssey Infrared Fluorescent Imaging System (LI-COR Biosciences). As a loading control, blots were also probed with either glyceraldehyde-3-phosphate dehydrogenase (GAPDH) or an actin antibody. Band densities were calculated using the integrated intensity values determined by the Odyssey software. Labeling was quantified by analyzing the ratio of the integrated intensity value of antibody-specific protein to the loading control in each lane to provide an integrated intensity ratio.

#### Antibodies

Primary antibodies (with the references provided where the antibodies were tested for specificity) included: metabotropic glutamate receptors [mGluR1 (AGC-006) [129] and mGluR5 (AGC-007) [24]] from Alomone (Jerusalem, Israel); phospholipase D [PLD1 (sc-25512) and PLD2 (sc-25513), [60]] and actin (sc-1616) from Santa Cruz Biotechnologies Inc. (Santa Cruz, CA); dopamine receptor D1R (AB20066) [130] from Abcam (Cambridge, MA); D5R (MAB5292) [131] from Millipore (Temecula, CA) and glyceraldehyde-3-phosphate dehydrogenase (GAPDH, Clone 6C5) from Advanced Immunochemicals Inc. (Long Beach, CA). Secondary antibodies included donkey anti-goat from Santa Cruz Biotechnologies Inc; goat anti-rabbit (926-32211-IRDye 800CW) and donkey anti-mouse antibodies (926-32222-IRDye 680) from LI-COR (Lincoln, NB).

#### PLD Activity Assay

PLD under normal conditions utilizes a molecule of water to catalyze the reaction generating phosphatidic acid (PA) and choline from phosphatidyl choline (PC). In the presence of simple alcohols such as ethanol, PLD preferentially utilizes the alcohol generating, in a 1∶1 ratio, phosphatidyl ethanol (PEtOH) [98]; which has been utilized as a quantitative enzymatic assay to measure PLD levels and activity. In addition to studying neuronal function with electrophysiology and measuring protein expression levels using western blots, we utilized this assay to study the enzymatic activity levels of PLD in the amygdala of animals following CPP and 14 day withdrawal. Animals were decapitated and 350 µm coronal brain slices were collected and placed in ice-cold (0–6°C) oxygenated ACSF. Amygdala subregions were dissected out from each brain slice by further making triangular cuts in the coronal slices containing the amygdala (see inset in Figure 7) and placed in test tubes containing 2 ml of Kreb's Buffer (pH 7.4) consisting of (in mM) NaCl (22); KCl (3.1); MgSO_4_ (1.2); KH_2_PO_4_ (0.4); and CaCl_2_ (1.3). After 30 min incubation at RT, Kreb's buffer was replaced with 1 ml ACSF containing 30 µCi of tritiated (^3^H) glycerol per sample tested, incubated for 2 hr and then washed with ACSF. Either 500 µl ACSF containing no ethanol, only ethanol (5 µl per sample), or ethanol and a drug were added in one to two slices per animal and incubated for an hour. Thus, every ethanol or ethanol and drug treated slice(s) had a control set of slices from the same animal that was not treated with ethanol or drug. The reaction was stopped by adding 2 ml of ice-cold chloroform/methanol/HCl (100/200/2) to each test tube. The tubes were then sonicated (30 min) and centrifuged at 4500 g for 2 min. The lower organic layer (1 ml) was collected and dried using nitrogen (N_2_) gas. Chloroform (70 µl) was then added to form the slurry and spotted on silica gel coated thin layer chromatography (TLC) plates. A solvent system consisting of ethyl acetate: 2,2,4–trimethyl pentane (iso-octane): acetic acid: methanol: water in 60:80:20:20:10 ratio was used to separate the components in the slurry. The plate was developed with iodine vapors and the resulting phosphatidylethanol (PEtOH) spot was visualized. PEtOH and other phospholipids (remaining in the lanes of each sample) were scraped separately from the plate and collected in individual vials of scintillation fluid to determine the ratio of PEtOH to total radioactivity counts (PEtOH/PLipids). Increases or decreases in PEtOH levels (calculated as percent of the control [no ethanol] levels per animal) were used to analyze changes in PLD activity.

#### Drugs

Cocaine HCl obtained from the National Institute of Drug Abuse (NIDA) was dissolved in 0.9% saline solution and injected intraperitoneally (i.p.) at a concentration of 15 mg/kg. Picrotoxin (PTX), dopamine (DA) and raclopride (RAC) from Sigma Aldrich (St. Louis, MO) were dissolved in water and kept on ice (prior to use). To prevent oxidation, fresh solutions were made immediately before experiments. 2-Methyl-6-(phenylethynyl)pyridine hydrochloride (MPEP HCl) was obtained from Ascent Scientific (North Somerset, United Kingdom). 6-Chloro-2,3,4,5-tetrahydro-1-phenyl-1H-3-benzazepine hydrobromide (SKF81297), (S)-(+)-α-amino-4-carboxy-2-methylbenzeneacetic acid (LY367385), (2R,1′S,2′R3′S)-2-(2′-carboxy-3′-phenylcyclopropyl)glycine (PCCG-13), 1-[6-[[(17b)-3-methoxyestra-1,3,5(10)-trien-17-yl]amino]hexyl]-1H-pyrrole-2,5-dione (U-73122), and R-(+)-7-chloro-8-hydroxy-3-methyl-1-phenyl-2,3,4,5-tetrahydro-1H-3-benzazepine hydrochloride (SCH23390) were obtained from Tocris Bioscience (Ellisville, MO). Ketamine HCl (Ketaved 100 mg/ml) and Xylazine HCl (Tranquived, 20 mg/ml) were obtained from Vedco Inc (St. Joseph, MO).

#### Statistical analysis

To account for non-normal distribution of data, non-parametric tests were used for statistical analysis. Behavioral data was analyzed using either one-way ANOVA (Kruskal-Wallis test) or a repeated measures two-way ANOVA followed by a paired or unpaired t-test when significance was achieved. Western blot and electrophysiological data were analyzed using a Kruskal-Wallis test followed by a Mann-Whitney U or Wilcoxon matched pair test as appropriate for pair-wise comparison. Statistical significance was defined at *p*<0.05, with an increasing number of asterisks indicating lower *p* values. Lack of significance (*p>*0.05) is denoted by “ns” (non-significant).

## Supporting Information

Figure S1 **Input-output relationships for fEPSP strength were not significantly altered in the BLA to lcCeA pathway after either saline or cocaine treatment compared to naïve group.** Responses are plotted for fEPSP strength (fEPSP slope, output) as a function of afferent BLA stimulation intensities (V, input). Slopes of the input-output curves were compared in three groups (naïve, saline-treated and 14 day withdrawn cocaine-cue CPP, n = 20–21 per group) with a Kruskal-Wallis ANOVA followed by pairwise comparison using Wilcoxon. Field EPSP slopes in slices from the amygdala of all three groups did not show any changes at different stimulation intensities tested.
(EPS)

Figure S2 **PCCG-13 blocks the expression of SKF81297-induced LTP.** Responses are plotted as percent change from the baseline fEPSPs as a function of time. SKF81297 (10 µM) application (for 15 min) in the presence of PTX ( µM) results in LTP (clear triangles measured in the 75–85 min duration, 150.4±6.9%, ***p<0.005, n = 4 compared to baseline) in amygdala slices from cocaine CPP animals while the saline-treated group (clear circles measured in the 75–85 min duration, 92.5±4.0%, ns, n = 4) exhibits no change in fEPSP. At 60 minutes after the ACSF mediated washout of the superfused SKF81297, PCCG-13 (2 µM) application (for 15 min) in the presence of PTX (10 µM) results in reducing the SKF81297-induced LTP to baseline values (clear triangles measured in the 150–160 min duration, 97.8±3.1%, ns, n = 4) in amygdala slices from cocaine CPP animals while no effect on the fEPSP response was observed in the saline-treated group (clear circles measured in the 150–160 min duration, 97.1±4.4%, ns, n = 4). Inset represents the fEPSP response in the cocaine CPP group at baseline (dark bar), last 10 min of the 60 min washout following SKF81297 (middle bar) and PCCG-13 (lightly shaded bar). Expression of SKF81297-induced LTP (150.4±6.9%, ***p<0.005, n = 4) was attenuated by application of PCCG-13 (97.8±3.1%, n = 4) compared to baseline (100.0±3.2%, n = 4). ***p<0.005 compared to baseline, ^###^ *p*<0.005 compared to fEPSP response after PCCG-13 application.
(EPS)
